# Upstaging and Downstaging in Gliomas—Clinical Implications for the Fifth Edition of the World Health Organization Classification of Tumors of the Central Nervous System

**DOI:** 10.3390/diagnostics13020197

**Published:** 2023-01-05

**Authors:** Oana Gabriela Trifănescu, Raluca Alexandra Trifănescu, Radu Mitrică, Dan Mitrea, Ana Ciornei, Mihai Georgescu, Ioana Butnariu, Laurenția Nicoleta Galeș, Luiza Șerbănescu, Rodica Maricela Anghel, Mihai-Andrei Păun

**Affiliations:** 1 Department of Oncology, “Carol Davila” University of Medicine and Pharmacy, 020021 Bucharest, Romania; 2 Radiotherapy II, “Prof. Dr. Al. Trestioreanu” Institute of Oncology, 022328 Bucharest, Romania; 3 Department of Endocrinology, “Carol Davila” University of Medicine and Pharmacy, 020021 Bucharest, Romania; 4 “C. I. Parhon” Bucharest Institute of Endocrinology, 011863 Bucharest, Romania; 5 Neuroaxis Neurology Clinic, 011302 Bucharest, Romania; 6 Department of Neurology, National Institute of Neurology and Neurovascular Diseases, 041914 Bucharest, Romania; 7 Medical Oncology II, “Prof. Dr. Al. Trestioreanu” Institute of Oncology, 022328 Bucharest, Romania

**Keywords:** glioblastoma, IDH status, WHO-CNS5, glioma, re-irradiation

## Abstract

In 2021, the 5th edition of the WHO Classification of Tumors of the Central Nervous System (WHO-CNS5) was published as the sixth volume of the international standard for brain and spinal cord tumor classification. The most remarkable practical change in the current classification involves grading gliomas according to molecular characterization. IDH mutant (10%) and IDH wild-type tumors (90%) are two different entities that possess unique biological features and various clinical outcomes regarding treatment response and overall survival. This article presents two comparative cases that highlight the clinical importance of these new classification standards. The first clinical case aimed to provide a comprehensive argument for determining the IDH status in tumors initially appearing as low-grade astrocytoma upon histologic examination, thus underlining the importance of the WHO-CNS5. The second case showed the implications of the histologic overdiagnosis of glioblastoma using the previous classification system with a treatment span of 7 years that proceeded through full-dose re-irradiation up to metronomic therapy. The new WHO-CNS5 classification significantly impacted complex neurooncological cases, thus changing the initial approach to a more precise therapeutic management.

## 1. Introduction

Gliomas, the most common primary adult brain tumors, have an annual incidence of 6 per 100,000 individuals; these rates are expected to increase by almost one-half in the upcoming 30 years. Of these, glioblastoma (GBM) is the deadliest and most frequent (53.5%) with an average survival of 15 months under the current standard of treatment (maximally safe tumor resection followed by the Stupp protocol), which is a significant improvement compared to the four months of survival without any postoperative treatment [1,2,3,4,5,6,7,8].

Accurate tumor classification is vital for correct diagnosis, accurate prognosis, and maximally safe treatment. In 2021, the fifth edition of the WHO Classification of Tumors of the Central Nervous System (WHO-CNS5) was published as the sixth volume of the international standard for brain and spinal cord tumor classification. The update followed the improved understanding of CNS molecular pathology and the recommendations of the Consortium to Inform Molecular and Practical Approaches to CNS Tumor Taxonomy (cIMPACT-NOW) [9]. One innovation in the current classification involves grading gliomas according to molecular characterization. Before the 2021 edition, the diagnosis of glioblastoma relied on histological findings of microvascular proliferation and necrosis and failed to discriminate between IDH mutant (10%) and IDH wild-type tumors (90%), which are two different entities that possess unique biological features and have different clinical outcomes with regard to treatment response and overall survival (OS) [8,10,11]. 

The molecular distinction between the mutant and wild-type IDH entities has been extensively studied for its impact on oncogenesis [12,13]. At first glance, these two entities appear to be two sides of the same coin because both imply enzymatic alterations of the Krebs cycle that affect critical metabolic processes, thereby leading to epigenetic changes in the DNA and thus collectively affecting gene expression, cell division, and differentiation [3,14]. However, IDH mutant tumors (most frequently through IDH1-R132H mutation) produce high levels of D-2-hydroxyglutarate (D-H2G), consequently promoting oncogenesis through the inhibition of key tumor suppressors. These processes disrupt DNA and histone methylation patterns and exhibit a signature of global DNA hypermethylation known as the Glioma CpG Island Methylator Phenotype, which can further promote the activation of oncogenes [15,16,17,18,19,20,21]. The metabolic reprogramming caused by D-H2G can induce further changes that promote gliomagenesis and render it more susceptible to radiation and chemotherapy [22,23,24,25,26,27,28,29]. In contrast to the IDH mutant tumors, the wild-type phenotype tumors overexpress IDH1, thereby leading to an excess of alpha-ketoglutarate, which can lead to epigenetic changes such as DNA and histone demethylation and metabolic changes that promote tumor progression and resistance to cell death, thus contributing to the tumor’s aggressiveness and resistance to treatment [12,30,31,32,33,34,35,36,37,38,39]. To conclude, there is an essential genetic distinction between IDH wild-type and IDH mutant tumors that can explain the difference in overall survival between the two (14 months for wild-type tumors versus 40 months for mutant tumors) and identify the crossroads in adult glioma diagnosis [3,40]. 

Therefore, the WHO-CNS5 classification illustrates the dichotomy between the two subtypes by implying that to confirm GBM, the IDH wild-type status is required and that all astrocytic tumors with IDH mutation will be excluded from being classified as GBM. It is also worth noting that astrocytic tumors with IDH wild-type status and three or more genetic parameters (such as TERT promoter mutation, EGFR gene amplification, or a combined gain of the entire chromosome 7 and loss of the entire chromosome 10 (+7/−10)) will be classified as GBM even in the absence of histologic features suggestive of GBM, thus supporting the superiority of molecular analysis over pathology examination [9,41,42]. 

Our overarching impetus was to showcase the importance of the new WHO classification and the new definition of GBM. Two seemingly similar cases, however very different in their outcomes, demonstrated the dichotomy between the IDH mutant and wild-type GBMs, highlighting the importance of determining the IDH status as a primary step in the diagnostic approach.

## 2. Case Presentation

This article presents two cases that highlighted the clinical importance of these new classification standards. One clinical case provided a comprehensive argument for determining the IDH wild-type status in tumors appearing as astrocytic on histologic examination, while the other aimed to show the pitfalls of histologic overdiagnosis of glioblastoma using the previous system of classification, thus underlining the influence of the WHO-CNS5. 

### 2.1. A Case of Grade 2 Diffuse Astrocytoma Upstaged to Glioblastoma

**Figure 1:**
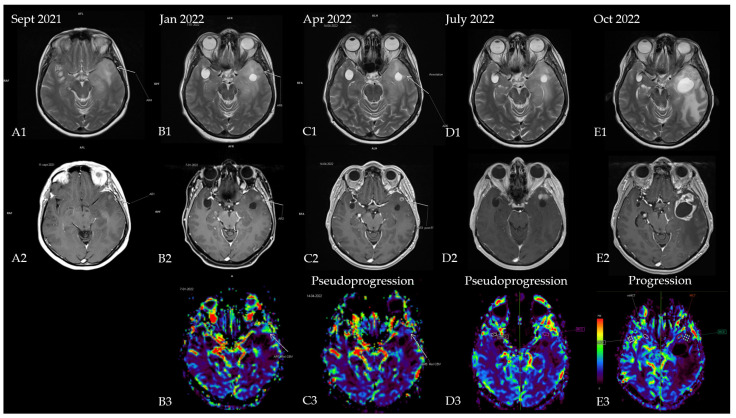
Patient’s disease progression showcased through serial brain MRI imaging. (**A**) Brain MRI at diagnosis ((**A1**)—T2 sequence showing diffuse hypersignal in the left temporal lobe; (**A2**)—T1 + contrast showing a slight nodular enhancement located in the anterior part of the temporal); (**B**) brain MRI after completion of the first PCV cycle ((**B1**)—T2 sequence with nodular hypersignal with CSF-like signal from the biopsy site; (**B2**)—T1 + contrast showing a slight increase in the size of the contrast-enhancing nodule; (**B3**)—postprocessing rCBV (relative cerebral blood volume) image showing hyperperfusion within the left temporal lobe). Results of the NGS testing corrected the initial diagnosis, and chemoradiotherapy was initiated. (**C**) Brain MRI 4 weeks after chemoradiotherapy ((**C1**)—T2 sequence showing mild reduction of the hypersignal; (**C2**)—T1 + contrast showing a nodular lesion with ring-shaped contrast enhancement with intralesional necrosis; (**C3**)—rCBV postprocessing showing reduction in perfusion in the enhancing area); (**D**) brain MRI showing pseudo-progression (3 months after starting TMZ and 4 months after completion of radiotherapy) ((**D1**)—T2 sequence showing mild reduction of the hypersignal; (**D2**)—T1 + contrast showing 2 nodular lesions with contrast enhancement; (**D3**)—rCBV postprocessing showing complete decrease in perfusion in the enhancing area); (**E**) brain MRI from October 2022 (1 month after she finished 6 cycles of TMZ and 7 months after radiotherapy completion) showing disease progression ((**E1**)—T2 sequence showing enhanced hypersignal and significant mass effect of a new lesion in the anterior temporal lobe with perilesional edema; (**E2**)—T1 + contrast showing a cystic lesion with ring-shaped contrast enhancement with intralesional necrosis and new extension within the anterior part of the temporal lobe; (**E3**)—rCBV postprocessing heatmap showing no apparent signs of hyperperfusion).

**Figure 2:**
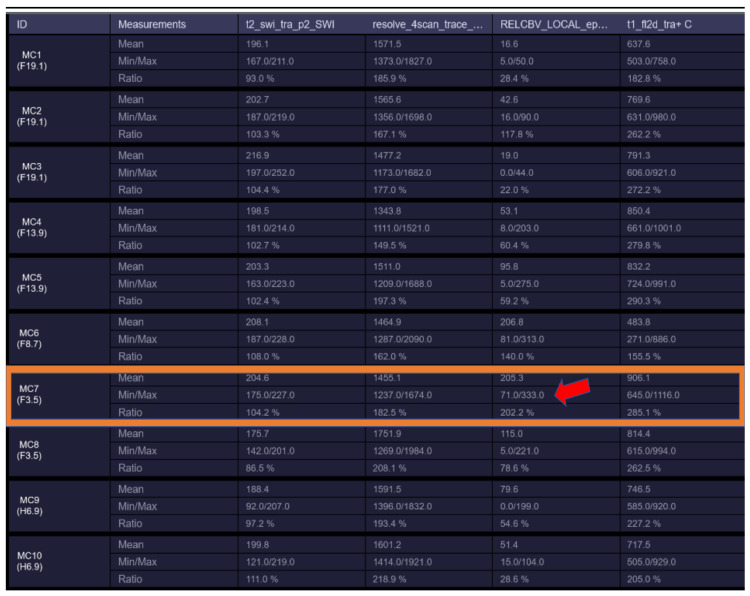
rCBV hyperperfusion indicating neoangiogenesis (MC7—showing rCBV > 1.7–1.9 threshold for neo-angiogenesis).

The first patient was a 53-year-old woman without any significant comorbidities who was admitted to a neurology clinic in September 2021 for loss of consciousness with sphincter incontinence and amnesia of the episode. Afterward, the patient expressed postictal right brachial paresis and mixed (predominantly expressive) aphasia that remitted 30 min after the episode. The patient described episodes of language disorder (which the patient interpreted as being correlated with stress) and insomnia several months before the episode. No focal signs of disease or abnormalities were observed in the neurological exam. A brain MRI revealed an infiltrative lesion in the T2/FLAIR hyper signal and T1 hypo signal occupying the left temporal lobe with left insular and thalamic extension, which was suggestive of diffuse glioma. Another lesion with a cystic appearance was discovered with a discrete T2/FLAIR hypersignaling area; this was most likely benign and representative of a perivascular space (Figure 1A1,A2). The patient was referred to neurosurgery for biopsy and symptomatic treatment with 8 mg of dexamethasone twice daily and 500 mg of levetiracetam twice daily was recommended. A percutaneous biopsy from the left temporal tumor guided by neuro-navigation was obtained, and multiple tissue samples were sent for pathological examination. A microscopic examination revealed infiltrative astrocytic proliferation with a low rate of cytonuclear atypia and rare mitoses arranged in high-density areas, which was associated with thin-walled blood vessels and mild perivascular chronic inflammatory infiltrate. Immunohistochemistry tests for ATRX, CD34, GFAP, Ki67 = 20%, and vimentin were positive, while p53 was negative, which was suggestive of diffuse astrocytoma (WHO Grade 2). In the absence of IDH1/2 testing, which at that time was unavailable in our country, the samples were sent for a next-generation sequencing (NGS) analysis to a neuropathology department in Paris (Pitié-Salpêtrière Hospital), and the patient was referred to our oncology clinic for treatment. Neoadjuvant chemotherapy based on the procarbazine, lomustine, and vincristine (PCV) regimen was administered for one cycle (from 27 October 2021 until 8 December 2021). NGS testing showed a negative IDH1 and a positive EGFRvIII mutation, which corrected the initial histopathology and immunohistochemistry results from diffuse astrocytoma (grade 2) to an early glioblastoma (grade 4) (the brain MRI at re-diagnosis is shown in Figure 1B1–B3. Consequently, concurrent radiotherapy (RT) with daily temozolomide (TMZ) (February 2022) was initiated followed by adjuvant chemotherapy with TMZ q5d/4w for six consecutive cycles. Treatment was monitored via serial brain MRIs conducted every three months (Figure 1C1–C3,D1–D3). After completion of the adjuvant chemotherapy, an MRI re-evaluation showed disease progression (Figure 1E1–E3 and Figure 2), and the patient decided against further treatment. The patient was still alive at the time of writing.

### 2.2. A Case of Glioblastoma Downstaged Due to the IDH Status

**Figure 3:**
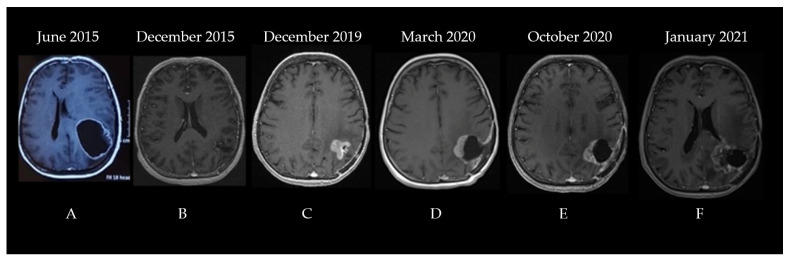
The patient’s disease progression showcased through serial brain MRI imaging ((**A**)—left frontoparietal process with a heterogeneous signal and structure, irregular margins, and both solid and cystic components surrounded by moderate edema, which compressed and tractioned the trigon and body of the left lateral ventricle upward, (**B**)—left parietal postoperative cavity with discrete wall contrast enhancement, minimal traction over the occipital horn of the left lateral ventricle, and midline structures in normal position, (**C**)—vascularized tumoral tissue on the medial and superior wall of the postsurgical resection cavity with perilesional edema, (**D**)—postoperative cavity and residual tumor after second surgery, (**E**)—postoperative cavity after second surgery with progression of residual tumor, (**F**)—further progression of residual tumor coupled with radiation induced changes).

The second patient was a 60-year-old woman diagnosed in June 2015. The patient was experiencing progressively worsening headaches and dizziness that debuted three weeks before her admittance to the neurosurgery clinic. The patient had no significant comorbidities; she underwent surgical resection for a lumbar herniated disc 10 years prior. A neurological examination did not show any abnormalities. A brain CT showed a space-occupying lesion in the left parietal lobe that was generating a mass effect on the ventricles. A subsequent MRI described a significant (56.5/51.3/50.2 mm in length/width/height) process situated in the left frontoparietal region with a heterogeneous signal and structure, irregular margins, and both solid and cystic components surrounded by moderate edema, which compressed and tractioned the trigon and body of the left lateral ventricle upward, thereby determining the displacement of the median line toward the right by approximately 10.5 mm (Figure 3A).

Consequently, the patient underwent surgical resection without postoperative complications with no residual contrast-enhancing tumor on postoperative imaging. The pathologic examination concluded that the cancer was a high-grade glioma with histological characteristics indicating GBM. Phenytoin at 100 mg three times/day was prescribed to control her seizures, and she was referred a month later to our clinic for adjuvant treatment. The brain MRI performed before radiotherapy showed a 21/20 mm left parietal postoperative cavity with discrete wall contrast enhancement, minimal traction over the occipital horn of the left lateral ventricle, and midline structures in a normal position (Figure 3B). The Stupp protocol was initiated: 3D conformal radio-chemotherapy to a total dose of 60 Gy (2 Gy/fraction for 30 fractions at 5 fractions/week) to the tumor bed and temozolomide administration (75 mg/m^2^ during radiotherapy) followed by adjuvant 5-day TMZ (200 mg/m^2^) every 28 days, which showed no significant adverse effects. After the adjuvant treatment, the patient was monitored every three months via brain MRI and clinical examination. The disease was clinically stable until December 2019. The MRI identified vascularized tumoral tissue on the medial and superior wall of the postsurgical resection cavity that measured 26/25 mm in axial diameters and 28 mm craniocaudal with perilesional edema (Figure 3C). Surgical reintervention on the parieto-occipital tumor was performed on 22 January 2020, and the pathology report identified a relapse of glioblastoma not otherwise specified. No further testing was performed, and the patient re-initiated therapy with TMZ 150 mg/m^2^ for two cycles followed by conventionally fractionated re-irradiation to a total dose of 60 Gy (2 Gy/fraction for 30 fractions at five fractions/week) to the tumor bed and residual tumor with concurrent TMZ (between 29 April and 16 June 2020) and adjuvant TMZ (June–October 2020) (see the pre-Stupp brain MRI shown in Figure 3D). The treatment was well tolerated. Following progression (identified through a re-evaluation brain MRI in October 2020—Figure 3E), a second-line therapy with lomustine (CCNU) at 90 mg/m^2^ was attempted for two cycles between November and December 2020. During the lomustine treatment, the patient developed a grade 3 thrombocytopenia in January 2021. After correction with corticosteroid treatment, it was replaced with carboplatin (CBDCA, which showed an area under the curve of 4 q3w) chemotherapy for seven cycles along with bevacizumab (15 mg/kg q3w) for the first four cycles (bevacizumab is not reimbursed for glioblastoma treatment in Romania, and the patient’s family could only afford the first four cycles of treatment). The brain MRI before initiating the carboplatin and bevacizumab regimen is shown in Figure 3F. 

**Figure 4:**
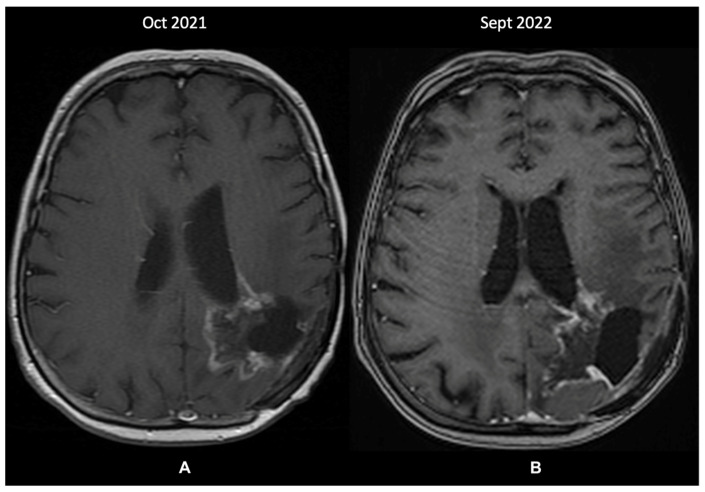
The patient’s last 2 MRI scans, T1 + contrast sequence (October 2021 and September 2022): (**A**)—changes within the left parietal lobe suggestive of recurrence and radiation-induced necrosis, extending medially from the postoperative cavity, around the subependymal space adjacent to the posterior horn of the left ventricle; (**B**)—discrete changes towards a probable tumor recurrence in the posterior part of the cystic cavity.

A re-evaluation brain MRI (October 2021—Figure 4A) identified further changes within the left parietal lobe that were suggestive of recurrence and radiation-induced necrosis. These measured 43/44 mm, extended medially from the postoperative cavity, and entirely circumscribed the subependymal space adjacent to the posterior horn of the left ventricle. Because the images showed structural progression of the contrast-enhancing lesions, without the possibility of undergoing a further biopsy, the patient was prescribed metronomic TMZ (50 mg/m^2^ daily). Since then, the disease has been clinically stable, and the last MRI in September 2022 showed minimal changes. Treatment was administered without any toxicity issues and was only interrupted for a brief period (two weeks) due to COVID-19 infection. However, compared to the previous MRI 11 months prior, a probable recurrence was visible in the posterior part of the cystic cavity (Figure 4B).

Considering the disease’s clinical evolution and treatment response, we considered re-challenging the initial diagnosis. We also sent pathology samples collected from the surgical re-intervention to Pitié-Salpêtrière Hospital in Paris for NGS analysis. The result was positive for an IDH1 R132H mutation (October 2022), thus correcting the diagnosis from GBM to an astrocytoma IDH mutant (grade 4). 

## 3. Discussion

This case review was intended to serve as a precise argument for the distinction between IDH mutant gliomas and IDH wild-type glioblastomas. Our cases were common with regard to the age, sex, and risk factors for gliomas: two female patients aged 53 and 60 years, respectively, with neither patient having any significant comorbidity or exposure to risk factors associated with gliomas [8,43,44,45,46,47]. Both patients had clinical manifestations consistent with the semiology described in the literature [46,48,49,50,51,52,53]. As in most cases, a brain MRI was the imaging standard that confirmed the clinical suspicion of brain neoplasia, and both patients presented typical MRI semiology for glioma [5,8]. 

The current standard for GBM treatment is maximally safe surgical resection that is continued with the Stupp protocol, which consists of concurrent RT and TMZ for six weeks followed by six months of adjuvant TMZ [1,2,8,54]. The initial therapeutic step in glioma treatment is the neurosurgical intervention aimed at mass effect relief, cytoreduction, and adequate tissue sampling for histologic and molecular tumor characterization [5]. Furthermore, it is well established that an extent of resection of over 90% (with a ≤5 cm^3^ residual non-contrast enhancing tumor) significantly improves the overall survival (OS) of GBM patients [8,55,56,57]. In our report, only the second patient was a candidate for tumor reduction, while the first patient was a candidate only for biopsy. However, a survival of over 14 months (the patient was still alive at the time of writing), which was close to the median survival of all treated GBM patients, was achieved with chemoradiotherapy, thus underlying the benefit of the Stupp protocol. 

According to the new classification, GBM is defined by histological features such as predominant nuclear and cellular atypia, a frequent mitotic index, necrosis, and vascular proliferation; as well as by molecular markers such as the IDH wild-type, the gain of chromosome 7 and loss of chromosome 10, amplification and rearrangements of tyrosine kinase receptors such as EGFR (50% of cases), aberrant telomere maintenance through TERT promoter mutations, alterations of the p53 pathway, and the mutation and deletions of PTEN (40%) [58]. On the other side, IDH mutant gliomas originate in low-grade tumors and show a progressive accumulation of genetic alterations and a progressive increase in the tumor grade. At the molecular level, an IDH mutation is associated with a loss-of-function mutation in tumor protein TP53 and ATRX [59].

In the light of WHO-CNS5 classification, the accurate detection of genetic variants such as single substitutions (IDH1/2 and TERT), chromosomal abnormalities (1p/19 q deletions, CDKN2A, and EGFR), or promoter methylation (MGMT) is critical for the correct diagnostic and treatment of gliomas. Next-generation sequencing (NGS) performed in reference genetic laboratories, rather than immunohistochemical assessment, is the most reliable method for evaluating the IDH status [42]. 

The pathology and molecular report that followed the tissue sampling represented the branch point between the two cases. 

Our first case was initially considered to be a grade 2 diffuse astrocytoma. In contrast, our second case was initially considered to be a grade 4 GBM following the microscopic and immunohistochemistry tests and according to the 2007 WHO Classification of Tumors of the Central Nervous System [60]. 

For the first patient, the molecular diagnosis change was pivotal because the neoadjuvant PCV chemotherapy (administered per current guidelines for diffuse astrocytoma WHO Grade 2) was stopped immediately after the first cycle, and the Stupp protocol was promptly commenced [2,8,61].

Regarding EGFR alteration in glioma, the most frequent is the deletion of exon 2–7 in the extracellular domain of EGFR, which results in the truncated mutant variant III (EGFRvIII); this was found in patient 1, which altered the prognostic and treatment plan. Activated EGFR may engage several signaling pathways that include PI3K/Akt, Ras/Raf/Mek/ERK, STAT3, and phospholipase C, thereby stimulating proliferation, invasion, angiogenesis, and resistance to apoptosis [62]. 

A recent study evaluated the prognostic factors and impact of treatment within molecular subgroups in 120 IDH wild-type former grade II diffuse astrocytomas and reported that patients with EGFR amplification had a worse outcome than those without EGFR amplification–median PFS 8 vs. 18.3 months and median OS 23.5 vs. 28.4 months compared to EGFR-intact subgroup (*p* = 0.040). No patient with an EGFR mutation was alive at five years, which emphasized the critical prognostic role [63]. 

In our second patient, the IDH mutant status could explain the dichotomy between GBM and astrocytoma (IDH mutant; WHO Grade 4). After gross total resection and the Stupp protocol, the patient had a prolonged progression-free survival (PFS). Even after relapsing, the disease was much easier to control by using full-dose re-irradiation and chemotherapy (the patient was still alive at the moment of writing—seven years after the initial diagnosis and three years after the disease progression was first documented) and showed an excellent response to both adjuvant and metronomic TMZ (PFS > 12 months for metronomic TMZ). 

There is no standard of treatment for recurrent glioma, and a multimodal approach consisting of surgical reintervention, irradiation, and multiple lines of chemotherapy is essential for obtaining a more prolonged survival in a glioma patient [54,64]. 

The data on the use of lomustine for recurrent GBM is controversial. In 1979, Hochberg published a retrospective study of the quality and length of survival of 74 patients treated with lomustine following craniotomy and irradiation. The results were discouraging and showed an mOS of 11.5 months (no benefit added compared to irradiation alone) but with an added benefit for the quality of life [65]. Phase III studies added further data on lomustine use for other drugs in the same setting but showed the same comparative results. It is also important to note that grade III-IV thrombocytopenia was a reported side effect [54,66,67,68]. 

The addition of bevacizumab to lomustine showed a significant improvement in oncologic outcomes in several studies by significantly prolonging PFS (4 months compared to 1 month for lomustine monotherapy). However, the combination did not confer a survival benefit over lomustine monotherapy, and as such, it is not reimbursed in many countries [54,69,70,71]. It is important to note that in our case, the lomustine + bevacizumab regimen was proposed to our patient as the first-line treatment for recurrent GBM. However, due to a lack of reimbursement and other drug access challenges, treatment with lomustine was started without bevacizumab and continued until intolerable toxicity occurred (grade III thrombocytopenia). Afterward, lomustine was switched to carboplatin, which was administered with bevacizumab, a combination that has been proven effective [72,73]. Despite the lack of OS benefit, the role of bevacizumab in recurrent GBM treatment might be underrated. Several studies have shown that the antiangiogenic effect of bevacizumab impacts the quality of life by limiting brain edema and radiation necrosis, which are very common in patients treated for glioma, especially in the setting of re-irradiation. As such, further evidence is needed to support the added benefit of bevacizumab in oncologic outcomes [54,74]

Metronomic temozolomide was tested in many phase I and II clinical trials with different results [75,76,77]. A study that included 37 patients with recurrent GBM and grade III glioma reported a 36% clinical control rate and a median overall survival of 7 months [76]. Worth noting is the exceptional compliance that the patient had under metronomic TMZ treatment; the administration was only postponed after a COVID-19 infection (the patient was unvaccinated for COVID-19) for two weeks [78]. In our institute, advanced-stage patients undergoing chemotherapy recorded increased mortality rates due to COVID-19 infection [79]. However, our patient managed to clear the infectious episode in good condition and resumed metronomic chemotherapy.

Our patient in the second case enjoyed a prolonged survival that was partially justified by the IDH mutant status. One consequence was the administration of two complete-dose radiotherapy cycles within a 5-year time frame. Although there are no treatment standards for a recurrent GBM, re-irradiation coupled with surgery is a frequently described local treatment option despite the increased risk of radionecrosis to previously irradiated brain tissues [80]. The optimal re-irradiation dose and radiotherapy technique is still a matter of debate. The cumulative EQD2 administered to GBM patients (the cumulative total dose from the two radiotherapy cycles normalized to 2 Gy fractions) was significantly associated with survival; cumulative doses ≥90 Gy provided a superior benefit compared to <90 Gy [81]. According to the literature, there is a 2–12% estimated risk of radiation necrosis at one year for cumulative EQD2  >  96.2 Gy and up to 17% for cumulative EQD2  >  137 Gy [80]. Concerning intracranial organs at risk, Niyazi et al. analyzed the long-term toxicity in 58 patients re-irradiated for malignant glioma and found no toxicity even at a cumulative EQD2 of 80.3 Gy, 79.4 Gy, and 95.2 Gy to the optic chiasm, optic nerves, and brainstem, respectively [82]. We considered our patient’s case to be favored by the intra-cranial parietal position of the tumor bed and residual tumor, which was relatively far from critical organs at risk, and the five years that elapsed after the first radiotherapy cycle; as such, a second 3D conformal, conventionally fractionated radiotherapy cycle was deemed feasible. In our patient’s case, the cumulative EQD2 was 120 Gy. Additionally, following re-irradiation, our patient completed four cycles of adjuvant bevacizumab, which may have proven to play a protective role because studies revealed its association with reduced treatment toxicity [83].

After a thorough review of the literature, we can also retrospectively remark on the considerable impact of the second surgery on our second patient. The re-intervention provided new tissue for histopathologic and biomarker analysis, prevented cranial hypertension, and positively impacted the patient’s outcome as shown by a 2021 study by Pasqualetti et al. [84]. 

These results aligned with data from the literature and highlighted the prognostic importance of the IDH status. In the future, new emerging biomarkers alongside IDH could improve the diagnostic and therapeutic management of GBM. A classic example would be the methylation of the O^6^-methylguanine-DNA methyltransferase (MGMT) promoter, which correlates to a better response to TMZ [85,86,87]. Other biomarkers include inflammation indexes (neutrophil x platelet x leukocyte/lymphocyte x monocyte—NPW/LM; or neutrophil x platelet x monocyte/lymphocyte—NPM/L) which proved to be valuable prognostic markers in a 2022 study [88]. Furthermore, biomarkers that predict the response to radiotherapy would be beneficial for managing GBM patients. Novel biomarkers such as the temporalis muscle thickness correlate to lower response and survival rates for radio- or radio-chemotherapy [89]. No less critical is the use of clinical prognostic factors (performance status, tumor dimension < 4 cm, gross total resection, and number of TMZ cycles), which is essential in order to stratify and delineate the following treatment options for the patient [90]. Further studies are needed to develop and validate new biomarkers to achieve the goal of a personalized and precise medicine. 

## 4. Conclusions

The WHO-CNS5 classification significantly impacted two complex neuro-oncology cases, thus changing the approach to a more precise therapeutic management. The scope of our case series was to provide a powerful argument for using the new WHO-CNS5 definition of GBM as the gold standard for diagnostic management in the era of precision medicine. The new classification provides a comprehensive redefinition of GBM while considering the evidence on the intrinsic differences between IDH mutant and IDH wild-type tumors. In the future, we expect to see IDH status determination in all GBM-suspected patients for diagnostic and prognostic purposes. We also look forward to seeing updated clinical data from emerging clinical trials that validate the differences in oncological outcomes of the distinct subgroups.

## Data Availability

The data presented in this study are available upon request from the corresponding author.
